# Teaching Children and Parents to Understand Dog Signaling

**DOI:** 10.3389/fvets.2018.00257

**Published:** 2018-11-20

**Authors:** Kerstin Meints, Victoria Brelsford, Tiny De Keuster

**Affiliations:** ^1^School of Psychology, University of Lincoln, Lincoln, United Kingdom; ^2^Department Nutrition, Genetics and Ethology, Faculty of Veterinary Medicine, Ghent University, Ghent, Belgium

**Keywords:** children, adults, dog body language, dog bite prevention, safety intervention

## Abstract

Safe human-dog relationships require understanding of dogs' signaling. As children are at particularly high risk of dog bites, we investigated longitudinally how children from 3 to 5 years and parents perceive and interpret dogs' distress signaling gestures. All participants were then taught how to link their perception of the dog with the correct interpretation of dogs' behavioral signals and tested again. Results show a significant increase in learning for children and adults, with them showing greater understanding of dogs' signaling after intervention. Better learning effects were found with increasing age and depended on the type of distress signaling of the dogs. Effects endured over time and it can be concluded that children and adults can be taught to interpret dogs' distress signaling more correctly. Awareness and recognition of dogs' stress signaling can be seen as an important first step in understanding the dog's perspective and are vital to enable safe interactions.

## Introduction

Benefits of dog ownership include positive effects on human health and well-being and on child development and learning [see (1) for overview; for recent systematic reviews, see (2, 3)]. Dogs function as social facilitators (4), assist in therapy, are used as co-visitors in retirement and care homes, in nurseries and in hospitals (1). Pets are seen as friends, companions and social partners (5–8) and, increasingly, as family members (5, 6). Dogs are among children's favorite pets and children show most attraction to dogs, be it puppies or grown-up dogs, compared to other pets (9, 10).

In the UK around 30% of households own a dog, with regional fluctuations in numbers (21–38%) (11–13), while in the US and in Australia up to about 40% of households own a dog (5, 14). The dog is also the pet of choice in many pet-owning households in Europe and Canada, with even higher figures in Mexico, Argentina and Brazil (15).

However, despite the benefits of dog ownership, there are also risks involved. Hospital data revealed that each year, about 1.5% of the general population suffers a dog bite that requires medical attention (16, 17) and the prevalence of dog bites in children is twice that of other age groups (18–20).

In the UK, a clear increase in the number of people attending a minor injury unit or accident and emergency department for treatment of dog bites and strikes has been observed. Over the ten-year period March 2005 to February 2015 the number of admissions due to dog bites increased 76% from 4,110 per year to 7,227. This is a 6.5% increase from the 6,783 finished admission episodes recorded in the previous 12 months (21). With the highest rate of dog bite injuries occurring in children (22–24), Schalamon et al. (25), demonstrated that most injuries occur in those under 15 years of age, with rates peaking between the ages of 5–9 years. Recent figures from the National Health Service on dog bites and strikes (21, 24, 26) demonstrate that more serious dog bite injuries requiring admission to hospital are on the increase, with 17% being related to children under the age of 10 years. Furthermore, dog bite rates in most-deprived compared to least-deprived areas are three times as high (21, 24).

However, the above estimate is low as these figures for adults and children do not include unreported cases were treatment was not required or where injuries were not presented to the medical profession (27, 28). Strikingly, when interviewed directly, about 47% of school children reported they had been bitten (28, 29). In a recent survey in the UK, Westgarth et al. (30) found that a quarter of their local sample of 694 adult respondents had suffered a dog bite.

High dog bite figures are not unique to the UK: the problem of dog bite injuries is a world-wide problem (31) with research from Australia (20), the Netherlands (23), Alaska (32), Belgium (33), Switzerland (18), Canada (34), and Spain (35) highlighting the extent of the issue. A recent study carried out by Quirk (27) estimated that 1,615,426 persons were treated in US emergency departments for non-fatal dog bite-related injuries between 2005 and 2009.

Costs caused by dog bite incidents are estimated at around $53.9 million for hospital stays only in the US (36), with home owners insurance claim payments reaching $530 million in 2014 (37). Likewise, costs in Australia were estimated around $7 million (38) and in the UK at around £10 million (39). Medical and veterinary professionals have repeatedly demanded effective prevention [e.g., (40)] and a collaborative (41) and evidence-based strategy (42, 43).

The majority of bite accidents (about 75%) occur in the home environment and involve children bitten by a familiar dog [e.g., (25), (44–47)]; see also (48) for similar data on adults]. Child-initiated interactions, such as approaching the dog while eating or surprising it while sleeping, seem to trigger up to 86% of accidents at home (44). Recent questionnaire studies also showed that injuries occurred during feeding treats or play (49).

Younger children are more often injured in the face, neck and upper torso (25, 46, 50). It has also been reported that 43% of patients on a maxillofacial ward for treatment after a dog bite were children under the age of 10 (40). Such injuries can lead to life-threatening medical conditions or psychological sequelae like Post-Traumatic Stress Disorder (51, 52). Whilst physical injuries are apparent, the psychological impact is less obvious, and left untreated can have long term consequences, not only for the victim but also their family (52). Seventy percent of all fatal dog bites involve children (53, 54).

Given these high figures, and given that most of the time, the child's interaction with a dog triggers the biting incident there is a clear need to increase parent awareness about home contexts and child actions that may trigger a dog bite (55, 56). There is also a need to improve the child's ability to assess how a dog responds to their action and for them to learn when it is not safe to interact with a dog. For appropriate supervision of children and dogs, it is also important for parents to be aware of the dog's signaling as reaction to their or their children's interactions with the dog.

Surprisingly, children as well as adults often do not notice dogs' stress signaling or misinterpret dogs' attempts to signal (57–59). When shown images of dogs' facial displays, children often do not understand dogs' facial expressions and can confuse a very angry dog as being friendly and approachable (60). Without tuition, children do not discriminate dogs' body signals and tend to look mainly at the face instead (61). In adults, dog signaling interpretations vary with experience, however, dog ownership does not predict correct understanding of dogs' behavior [e.g., (62, 63)].

Overall, research has demonstrated that there is little knowledge regarding dog behavior and safety practices for child-dog interactions [see also (64, 65)]. When trying to enable safe human-animal interaction, it is vital to be able to recognize and interpret the animal's distress signaling correctly in order to avoid injury to the person and distress to the animal. Arhant et al. (49) also emphasize the need for a dog bite prevention approach directed at caregivers.

While dog bite prevention programmes exist, and some address how to behave in public with unfamiliar dogs [e.g., (18, 66, 67), see (68) for a systematic review], while others teach children and their families to be aware of potential risk situations with a family dog, and how to avoid or de-escalate risk situations [e.g., Blue Dog bite prevention program assessment; see (56, 69)] there is no assessed program so far that teaches children or adults more basic skills—how to recognize and interpret specific dog body language. More precisely, currently no intervention has been tested to teach children and adults about dogs' behavioral response and their stress signals as a response to the child or adult in the context of a dog-directed action.

Humans often perceive petting a dog or hugging a dog as friendly gestures. Especially young children like to hug dogs as a sign of their friendship, not realizing that their (benign) actions might intimidate a dog and induce fear or distress. If a dog freezes and does not move, this may lead parents and teachers to think the dog feels happy with this well-intended attention. Thus, when targeting dog bite prevention in families with children and their pet dog, it is crucial to realize that safe cohabitation is based on mutual understanding of species-specific signaling, social gestures and interactions (70). Research indicates that most of the dog bite accidents with family dogs result from such seemingly benign (from the human perspective) interactions, hence the importance to stimulate awareness in children and parents about how their dog behaves, and which signals the dog presents when being hugged, petted or approached in different situations (55). Recent research has shown that most of children's interactions with dogs fall into this category, and mostly increase in frequency with age (49).

Dogs who feel stressed are likely to present stress- and threat-avoiding signaling (e.g., nose-licking, turning away). When these signs are ignored or misinterpreted, the pet may use other strategies, including aggression [(71–73); see also Mariti et al. (74) for a first systematic empirical investigation of such behaviors in dogs]. Recent studies have shown further evidence that dogs show signals like licking of lips and looking away as appeasement signals in dog-human communication [(75); see also (76)].

**Figure 1:**
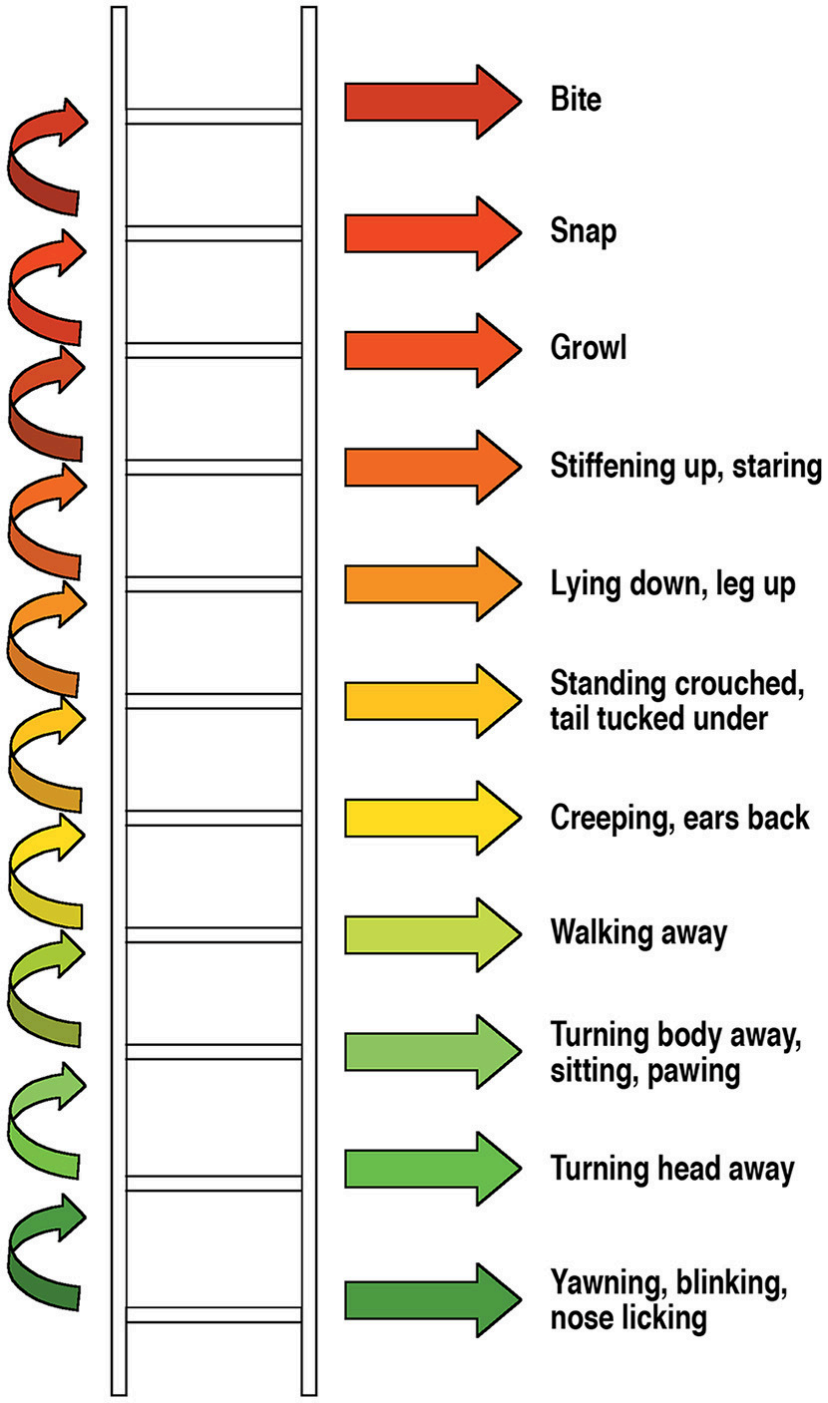
How a dog can react to stress or threat, Shepherd (2002, 2009), used with permission from BSAVA Manual of Canine and Feline Behavioural Medicine, 2nd edition (2009).

Shepherd's “ladder” of distress signals (72, 73) includes *conflict-defusing signals* on its lower steps (appeasement behavior, calming signals, displacement behavior, e.g., nose-licking, eye-blinking)—these are signals to defuse conflict and restore harmony in a social interaction. In the next grouping on the ladder, *conflict-avoiding signals* are included (e.g., walking away, standing crouched, tail tucked under, creeping). In case a perceived social threat continues, and/or conflict-defusing avoiding strategies have failed, dogs may present strategies higher on the ladder such as *conflict escalation signals* (e.g., staring, growling, biting). For an overview, see Figure 1.

It is important to stress that Shepherd's ladder is not to be understood in a strictly hierarchical way as dogs do not necessarily move through these signals in a linear fashion. Depending on how the interaction evolves (i.e., if the approaching human understood the message correctly, and by stopping all interaction with the dog, the dog may be able to relax and return to a state of comfort) and depending on what the dog has learnt (e.g., unpleasant outcome of interactions in the past despite conflict-avoiding signaling), their strategy may change over time, and dogs may move on to a snap or bite action to stop a perceived threat.

It is also vital to be aware that dogs' strategies depend on factors relating to the context (social & environmental triggers), factors relating to the dog e.g., personal history (past experiences) and on their physical and behavioral health. It is important to stress that factors that are known to reduce a dog's wellbeing will reduce a dog's threshold for stress and arousal and increase the odds of using escalation strategies in a stressful encounter. Well-known examples are sensory deficits, physical illness, chronical pain or dogs suffering from anxiety (45, 47, 77). In addition, other signals may be shown [e.g., (57, 71, 74)].

There is a striking lack of knowledge of dog signaling in the population, and there is also a general lack of knowledge regarding dog behavior and safety practices for child-dog interactions, with owners of dogs often unaware of the factors likely to increase the risk of dog bites to children (64), for example, subtle signals are often not known by dog owners to be stress signals (58). This is a serious knowledge gap, as the safety of young children mainly relies on the perceptual understanding, and knowledge and anticipatory guidance of the adults around them (47, 64). The following steps are often named to constitute a more complete process of prevention and action:

Step 1 *Knowledge* of stress signaling
Step 2 Recognition and correct interpretation of stress signaling
Step 3 *Adapt the action*: awareness of the situation and insight to act accordingly
Step 4 *Repeat*- *Recognition* of future contexts and avoidance of risk (78).

Thus, while dogs are rather good at interpreting human signaling [e.g., (79–92)], humans do not seem to be equally equipped to interpret dog's visual signaling.

Given not only the popularity of dogs as pets, also the increasing popularity of animal-assisted interventions in educational settings as well as the application of pets in the classroom [(93); for a systematic review, see (2); see also (1, 94–99)], and given the frequency of injury with familiar dogs at home, there is an urgent need to teach adults and children dog body language.

In order for children to interact safely with dogs, they must first have knowledge of dog behavior and awareness of situations which may put them at risk of being bitten. This means that they must know the signals, recognize them, understand that they are the consequence of actions toward the dog, and, if it is their own action, adapt their action. Ultimately, it is crucial that parents also have this knowledge in order to teach and supervise their children when interacting with dogs and to provide anticipatory guidance.

If we can successfully teach children and parents to recognize and interpret dogs' stress signaling correctly, and be aware of the actions that trigger the signaling, and ideally, act upon their knowledge, then all sides will profit: adults and children will understand dogs' distress signaling better, risk situations may be defused and the (family) dog will enjoy more respectful and appropriate treatment.

In the current study, we have addressed the lack of knowledge and lack of systematic intervention with children and adults alike. By teaching participants how to recognize and interpret dog stress signals and by assessing if our intervention works, we are undertaking the first steps toward preventing misunderstandings and risk escalation due to lack of knowledge.

We assessed participants' knowledge of dogs' signaling behaviors before and after a dog body language intervention with a range of video clips of real dogs. We tested both children and parents. In addition, we integrated this into a longitudinal design to monitor the effectiveness of the current intervention by assessing children's developmental progression over 4 time points up to 1 year. Finally, to gain more in-depth knowledge of other potential factors, we used questionnaires to learn about background demographic data, socio-economic status and dog ownership statistics.

## Methods

### Participants

Children were recruited through schools and nurseries in the county of Lincolnshire, UK. All participants were healthy and had normal or corrected-to-normal vision. No exclusions occurred before testing.

Initial calculations with Poweranalysis [G^*^Power3; (100)] showed a necessary sample size of 18 children per age group (3, 4, and 5 years). As attrition rates of about 30–70% do occur in longitudinal studies and often reduce the initial cohort to a vastly smaller size in the final cohort (101), we over-recruited children to be able to cope even with a harsher drop-out rate. Hence, our initial overall group size at Test 1 contained 124 children for this longitudinal study. However, our attrition rate was very low and we managed to keep 82% (*N* = 101) of children in the sample after 6 months and we retained 85% (*N* = 105) of children in the final sample after 1 year as can be seen in the following Table 1.

**Table 1:** Participant numbers over time.

	**Test 1 (baseline)**	**Test 2 (same day)**	**Test 3 (6 months later)**	**Test 4 (1 year later)**
Age groups at start	124 children (N, mean, range and SD)	121 children	101 children	105 children
3 years	*N* = 44 (17 females, 27 males, mean age = 3.4, age range; 2.8–3.9, *SD* = .3)	*N* = 42, (17 females, 25 males, mean age = 3.4, age range; 2.8–3.9, *SD* = .3)	*N* = 31 (12 females, 19 males, mean age = 3.9, age range; 3.4–3.9, *SD* = .4)	*N* = 34, (15 females, 19 males, mean age = 4.5, age range; 4.0–5.1, *SD* = .3)
4 years	*N* = 31 (15 females, 16 males, mean age = 4.6, age range; 4.0–4.9, *SD* = .2)	*N* = 30 (14 females, 16 males, mean age = 4.6, age range; 4.0–4.9, *SD* = .2)	*N* = 29 (14 females, 15 males, mean age = 5.0, age range; 4.4–5.4, *SD* = .2)	*N* = 24 (11 females, 13 males, mean age = 5.6, age range; 5.2–5.9, *SD* = .2)
5 years	*N* = 49 (23 females, 26 males, mean age = 5.7, age range; 5.0–6.8, *SD* = .4)	Same as at Test 1	*N* = 61 (18 females, 23 males, mean age = 6.2, age range; 5.5–7.3, *SD* = .5)	*N* = 47 (22 females, 25 males, mean age = 6.0, age range; 5.8–7.8, *SD* = .5)
Adults	40 parents (8 males, 32 females, mean age = 38.9 years; *SD* = 4.9)	n/a	n/a	n/a

Children took part in Test 1, 2, 3, and 4. Reasons for attrition in children are as follows: In Test 2, 3 children who took part in Test 1 did not complete Test 2 on the same day, hence were excluded from analysis. Test 3: Attrition of 20 children due to being ill, having moved school and being on holiday. Test 4: A slight gain of children occurred, as some who had missed Test 3 due to absence were back for Test 4.

Overall, in the final sample entered into the data set, there are 88 children who took part in all testing sessions (39 girls and 49 boys overall; 26 3-year-olds (12 = female, 14 = male; *M* = 3.4, *SD* = .32, range 2.8–3.9), 23 4-year-olds (11 female, 12 male; *M* = 4.6, *SD* = .24, range 4.0–4.9) and 39 5-year-olds (16 female, 23 male; *M* = 5.7, *SD* = .45, range 5.0–6.8). Of this sample, 37% had a dog.

Parents took part in Test 1 and 2 (same day) only. Additional longitudinal parent testing was not possible due to limited funding. However, piloting had shown that adults showed clear improvements as they found the teaching phase to be a real “eye-opener.” Error rates dropped once they had realized what the behavior of the dog implied. The current study results confirm this and we have no reason to assume that adults with typical and intact memory capacity would forget this knowledge over time. Of the parents 27.5% were dog owners, these dog ownership figures for children and adults compare well with the national average of about 30% dog owners. Also, 47.5% of parents had been bitten by a dog, this is very similar to the 47% reported elsewhere [e.g., (29)]. Thus, we can assume our sample is fairly representative concerning these factors.

#### Ethical approval

This study was carried out in accordance with the recommendations of University of Lincoln, School of Psychology Research Ethics Committee (SOPREC). The protocol was approved by the SOPREC. Written informed consent was gathered in accordance with the Declaration of Helsinki.

### Stimuli

#### Video clips

The stimuli consisted of sets of 16 short video-clips portraying dogs with the full range of behavioral distress signals described in “Shepherd's ladder” (72, 73). These are as follows: yawning, blinking, nose licking, turning the head away, turning the body away, pawing, walking away, creeping, crouching with tail tucked under, lying down with legs up, stiffening up and staring, growling, snapping and biting. Due to other literature, we also added snarling and walking away with hiding. We also presented four video clips of relaxed dogs. Given research indicating that acoustic input may help children's recognition and correct interpretation (102, 103), those clips that naturally had a sound (snarling and growling) were accompanied by this sound.

Due to ethical considerations we did not show serious bites drawing blood, and parents had the opportunity to view the images beforehand and decide if they allowed their children to take part. Also, children received a thorough debriefing session after testing finished, so we could make sure children clearly understood the dog signaling. None of the children displayed any signs of distress during testing or after testing, none of the parents reported any detrimental effects back to the research team.

Having piloted the video clips, we decided that the procedure worked best if we used 2 × 16 videos (2 per distress behavior)—we added 4 relaxed (happy) behaviors so that children would not get the impression that dogs are usually distressed, however, these items were not part of the intervention phase and children were not trained to recognized relaxed dogs^1^. We called these “happy” as the language needed to be child-appropriate and previous work has shown that children understood this label well, similar for the terms “ok,” “unhappy,” and “angry” (60).

Videos were clipped and resized using Bink and Smacker (RAD Video Tools): each video was 6,000 ms duration, 360 × 240 pixels, and with a data rate of 25 frames per second.

Video clips were presented centrally on the monitor screen and displayed on a 15% greyscale background. Altogether, we used up to 4 different sets of videos in Test 1 (baseline), Test 2, 3, and 4 (see below). All video stimuli were assessed for their expression and approved by 3 internationally renowned dog behavior specialists.

#### Audio stimuli

Audio recordings matching each of the visual stimuli were produced in a sound-proof professional audio-recording studio at the University. All recordings were carried out within one session so as to reduce variation in the voice of the speaker. The speaker was female and a native speaker of British English. Audio messages consisted of four features across all trials: an initial “Look” command, followed by a description of the dogs' behavioral signal to steer participants' attention, then a message of how the dog is feeling and lastly a message of safety instruction for the child. We have consulted closely with a consultant and dog behavior expert on the appropriate content of the verbal messages. Messages take the following character: (a) Attention getter (Look!), (b) highlighting the dog's signaling behavior, (c) followed by an explanation how to interpret the dog's behavior, (d) then a clear safety instruction for adapting their actions. An example of such a message is as follows: “Look! The dog is blinking its eyes. The dog is worried. You should leave the dog alone.” Audio files were cut and manipulated using Audacity version 2.0.1. Files were 1141 kbps, 2 channel and were used in .wav format.

#### Rating scale

We used a child-appropriate 1-5 rating scale in which symbolic faces expressed either very happy (1), happy (2), just ok (3), unhappy/angry (4), and very unhappy/angry (5) emotions. Children had no problems using the scale.

### Procedure

Children were tested in schools and nurseries in a quiet room. Videos were presented on a laptop and the experiment was programmed using the Lincoln Infant Lab Package 1.0 (104). Participants were seated approximately 70 cm from the screen.

Child participants took part in the study longitudinally; this included viewing an initial baseline phase of video stimuli (Test 1), immediately followed by a training phase of videos and then tested with novel videos (Test 2) afterwards to investigate if their knowledge had improved. Participants were then tested again 6- and 12 months later (Test 3 and 4) without any additional training to see if they had retained their knowledge. Hence, we have an integrated control group with each child being their own control (before and after learning and at the follow-up testing). In addition, we have further integrated controls in that 4-year-olds at testing start can be compared with 3-year-olds after 1 year (when they have turned 4 years of age). In the same way, the 5-year-olds at start of their testing can be compared with the 4-year-olds at testing point 1 year (when they have turned 5). Adults only took part twice on the same day (Test 1, Training and Test 2) and results can therefore be compared before and after testing.

#### Testing phases

##### Baseline phase

Each participant viewed 20 trials. Each trial was made up of a 6,000 ms video displaying dog behavioral signals as described above. These were followed by a fixed choice user/child friendly rating 1–5 scale ranging from “very happy” to “very unhappy/angry.” Participant ratings were recorded both electronically and verbally, and the rating scale stayed on the screen until the participant had made their choice. Duration of this phase was between 2 and 5 min.

##### Training phase

Participants viewed 32 trials (2 × 16 distress behaviors, one set with dogs seen in Test 1, one set with novel dogs). Each trial was made up of a 1,000 ms blank screen accompanied by the initial “Look” audio. This was followed by a 6,000 ms video displaying dog behavioral signals accompanied by the remainder of the audio sentence highlighting the dogs' behavioral stress. Duration of this phase is about 4–5 min.

##### Test 2 (same day) and test 3 and 4 (6- and 12-month intervals)

Participants were again presented with 20 trials (16 distress behaviors and an additional 4 “happy” dogs). This was immediately followed by the fixed choice user/child friendly rating 1–5 scale as described above. This took between 2 and 5 min. Both, children and parents thoroughly enjoyed taking part.

Note: In addition, half of the children always saw novel stimuli at each testing time, and the other half saw the novel set from Test 2 repeated at Tests 3 and 4. This was to explore if children learn differently with items that are novel each time as opposed to items that are novel at Test 2 and then reoccur, however, there was no statistical difference, hence, results below include both groups of stimuli.

## Results

### Study 1 with children

#### Rating scores children

We initially calculated a repeated measures ANOVA with Gender (male/female), Dog Ownership (yes/no), Age Group (3, 4, 5 years) and Distress Signal Group (defuse, avoid, escalate) on the rating scores at different Testing times (before training, after training, after 6 months, after 1 year)^2^. This analysis revealed no significant effects of Gender and Dog Ownership, hence we calculated a repeated measures ANOVA only with Age Group (3, 4, 5 years) and Distress Signal Group (conflict defusing, conflict avoiding, conflict escalating) on the rating scores at the different testing times (before training, after training, after 6 months, after 1 year).

We found a highly significant main effect of Age [*F*_(2, 85)_ = 7.84, *p* < .001, partial η^2^ = .16] with older children showing more correct results than younger children. A significant main effect of Distress Signal Group [*F*_(2, 170)_ = 298.85, *p* < .001, partial η^2^ = .78] also emerged, with children judging conflict escalating signals as different from conflict-avoiding and defusing signals, but not distinguishing between conflict-avoiding and defusing signals in dogs–*post hoc* tests with Bonferroni corrections (*p* < .0166) show that the following differences are highly significant: conflict-escalating vs. conflict-defusing (*p* < .001); conflict-escalating vs. conflict-avoiding (*p* < .0001); while children do not distinguish conflict-defusing vs. conflict-avoiding signals in dogs (*p* < .05).

We also found a highly significant main effect for Testing times [*F*_(3, 255)_ = 6.93, *p* = .0002, partial η^2^ = .08] with children improving significantly from Test 1 (baseline measure before intervention) to Test 2 after intervention (*p* < .002). Children also show improved knowledge from Test 1 to Test 3 at 6 months (*p* < .0026) and from Test 1 to Test 4 after 1 year (*p* < .0006).

**Figure 2:**
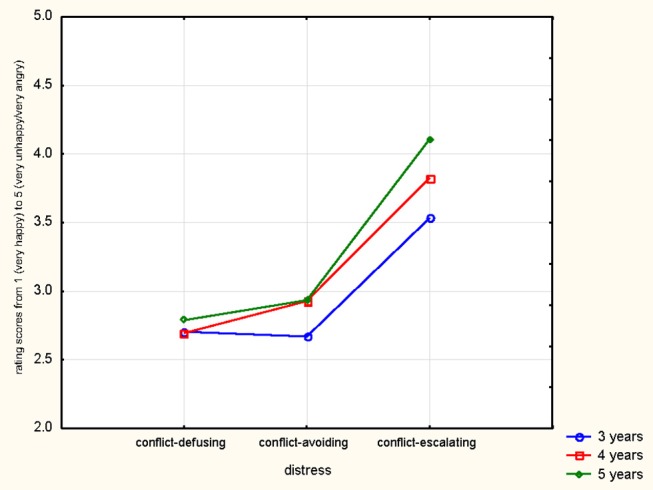
Rating results shown for distress signal group and children's ages.

There was also a significant interaction between Age group and Testing Times [*F*_(6, 255)_ = 5.11, *p* = .0001, partial η^2^ = .11] which demonstrated that the older the participants, the better they perform. Highly significant interactions of Age by Distress Signal [*F*_(4, 170)_ = 5.07, *p* = .0007, partial η^2^ = .11], see Figure 2 below, and of Distress Signal by Testing Time [*F*_(6, 510)_ = 6.02, *p* < .0001 partial η^2^ = .07] also emerged as well as a significant three-way interaction between Testing time, Distress Signal and Age [*F*_(12, 510)_ = 1.94, *p* = .028, partial η^2^ = .04] showing clear differences between conflict-escalating signals vs. conflict-defusing and avoiding signals, with children showing better performance with increasing age and improvement over time, especially in the conflict-escalating signal group.

**Figure 3:**
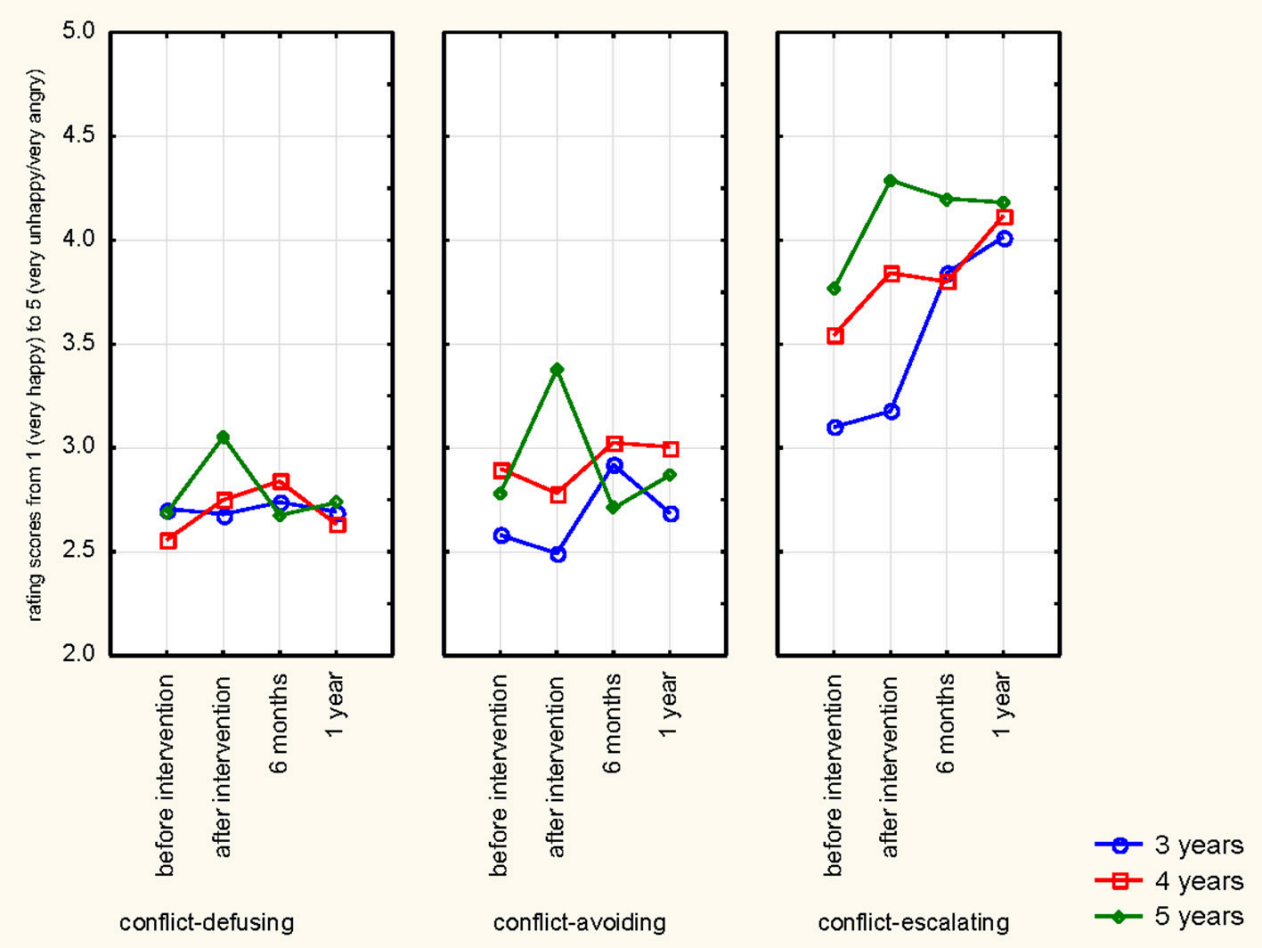
Overview of all results: Rating results for distress signal group and children's ages over time.

These results show medium to high effect sizes. Results are illustrated in overview in Figures 2, 3.

After the intervention, children improve in their judgments, but even the oldest children do not come close to the correct ratings (e.g., 5 for conflict-escalating signal, 1 for happy).

### Study 2 with adults

#### Rating scores adults

**Figure 4:**
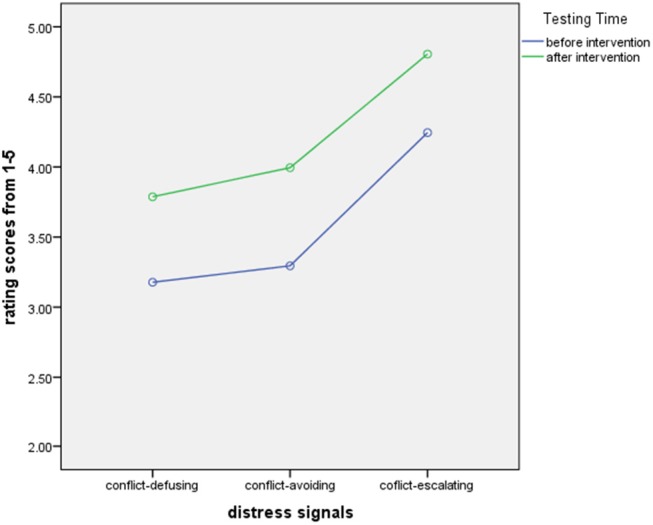
Rating scores for adults by distress signal group before and after intervention.

An ANOVA of Gender (male/female) by Dog Ownership (yes/no) by Distress Signal group (conflict-defusing, conflict-avoiding, conflict-escalating) was calculated for Testing Times before and after intervention on rating scores. Gender and Dog Ownership yielded no significant results, therefore the analysis was calculated with Distress Signal group (conflict-defusing, conflict-avoiding, conflict-escalating) and Testing Times before and after intervention. We found a highly significant main effect for Testing Time [*F*_(1, 39)_ = 243.93, *p* = .0001, partial η^2^ = .86] showing improved understanding after intervention and a highly significant main effect for Distress Signal group [*F*_(2, 78)_ = 291.54, *p* = .0001, partial η^2^ = .88] highlighting differences between Distress Signal groups. Figure 4 below illustrates this.

After the intervention, adults come close to the ratings that would be suitable for the dog's signaling attempt (5 for conflict-escalating signals, 4-4.5 for conflict-avoiding signals, 4 for conflict-defusing signals).

We also tested if there were effects for parental education, but no significant results existed.

### Studies 1 and 2: rating scores compared

#### Children and adults

We also found highly significant main effects on differences between the parents' and children's judgments of dog's behavior, with most mistakes occurring in the conflict-defusing and conflict-avoiding signal groups [*F*_(3, 387)_ = 251.69; *p* < .0001].

#### Expected vs. obtained scores

One-sample *t*-tests revealed that all age groups significantly underestimate and misinterpret the dogs' real distress signaling (*p* < .001). Again, younger children make most misinterpretations. Least recognition of different distress signaling is found in 3-year-old children.

### Studies 1 and 2: correct answers and errors

In a further analysis, we calculated correct responses and errors from the original scores. Table 2 below shows percentages of correct answers and errors per Distress Signal category^3^. Please note the high proportion of errors classed as “happy” by the participants.

**Table 2:** Correct answers and errors in % over time for children and adults.

	**Pre-training**		**Post-training**		**After 6 months**		**After 1 year**	
	**Correct**	**Error**	**Correct**	**Error**	**Correct**	**Error**	**Correct**	**Error**
**CONFLICT ESCALATING SIGNALS**								
3 years	47%	53% “happy” 65%	50%	50% “happy” 58%	64%	36% “happy” 56%	66%	34% “happy” 58%
4 years	55%	45% “happy” 50%	72%	28% “happy” 69%	70%	30% “happy” 62%	76%	24% “happy” 41%
5 years	64%	36% “happy” 52%	83%	17% “happy” 43%	77%	23% “happy” 38%	81%	19% “happy” 36%
Parents	83%	17% “happy” 16%	100%	–				
**CONFLICT AVOIDING SIGNALS**								
3 years	23%	77% “happy” 68%	26%	74% “happy” 75%	33%	67% “happy” 49%	30%	70% “happy” 62%
4 years	31%	69% “happy” 51%	27%	73% “happy” 58%	33%	67% “happy” 49%	36%	64% “happy” 37%
5 years	27%	73% “happy” 56%	42%	58% “happy” 34%	25%	75% “happy” 51%	20%	80% “happy” 57%
Parents	52%	48% “happy” 66%	93%	7% “happy” 36%				
**CONFLICT DEFUSING SIGNALS**								
3 years	16%	84% “happy” 54%	14%	86% “happy” 64%	16%	84% “happy” 50%	14%	86% “happy” 58%
4 years	13%	87% “happy” 55%	13%	87% “happy” 50%	15%	85% “happy” 45%	17%	83% “happy” 45%
5 years	20%	80% “happy” 56%	20%	80% “happy” 35%	18%	82% “happy” 44%	13%	87% “happy” 41%
Parents	28%	72% “happy” 14%	73%	27% “happy” 16%				

*Based on 114 children overall and 40 adults*.

#### Correlations between children's and parents' responses

There were no significant correlations between children's and their parents' judgments of the dogs' signaling behaviors before or after training.

#### Correct answers and errors – children

**Figure 5:**
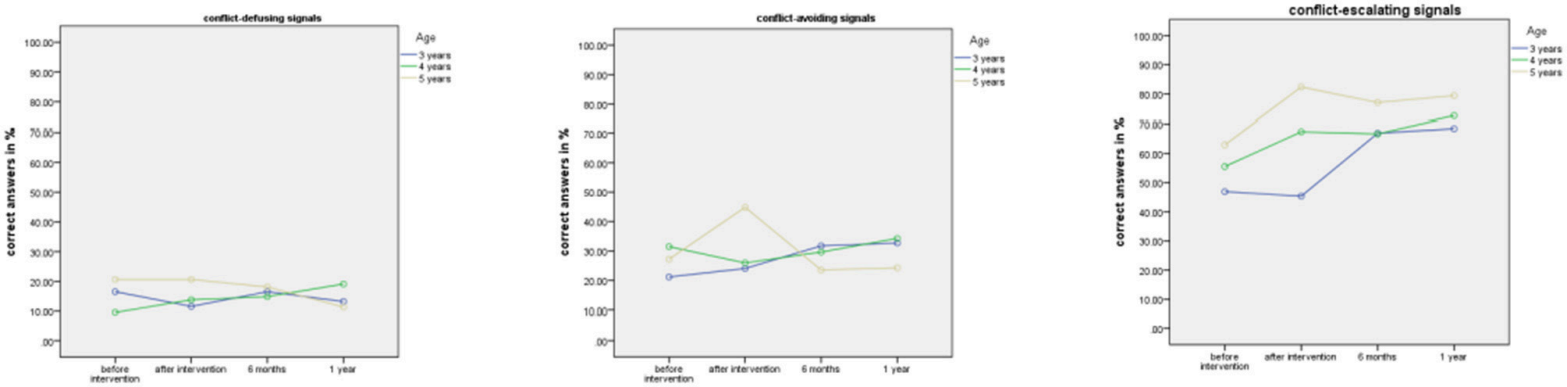
Results in overview for children by signal group before and after intervention, at 6 months and 1 year.

We also calculated a repeated measures Anova of Gender (male/female) by Dog Ownership (yes/no) by Age Group (3, 4, 5) by Distress Signal Group (conflict-defusing, conflict-avoiding, conflict-escalating) before and after Intervention (Test 1, 2, 3, and 4) on correct answers. As there were no effects of dog ownership or gender, we ran the analysis with Age Group (3, 4, 5) by Distress Group (conflict-defusing, conflict-avoiding, conflict-escalating) at the different testing times (before training, after training, after 6 months, after 1 year). The following main effects were found: A significant main effect for Age [*F*_(2, 148.822)_ = 6.98, *p* = .002, partial η^2^ = .14] and Distress Signal Group [*F*_(1.772, 84)_ = 395.36, *p* = .0001, partial η^2^ = .83] as well as Testing Time [*F*_(2.823, 237.156)_ = 4.72, *p* = .004, partial η^2^ = .053]. Significant interactions were shown for Testing Time by Age [*F*_(6, 84)_ = 4.94, *p* = .001, partial η^2^ = .11], Distress Signal by Age [*F*_(4, 84)_ = 4.298, *p* = .002, partial η^2^ = .93] and Testing Time by Distress Signal [*F*_(5.643, 473.980)_ = 4.70, *p* = .001, partial η^2^ = .53]. Overall, children distinguish conflict-escalating signals better than conflict-avoiding and conflict-defusing signals. They show more correct answers with increasing age and improve after intervention, specifically in the conflict-escalating signal group. In this group, improvements are stable over time (up to 1 year). The 5-year-olds also improve in the conflict-avoiding signal group from before to after intervention, however, this effect is not enduring over time. Interestingly, despite the same rating categories 4 and 5 accepted for conflict-avoiding and conflict-escalating signals, children distinguished conflict-avoiding and conflict-escalating signals clearly (*p* < .0001). Overall, these results show significant differences over time and for the different distress groups, with older children giving more correct answers than younger children. See Figure 5 below for an overview of the results.

Concerning the question if children just learn over time or if results are due to our intervention, we have compared results of children at 4 and 5 years (4-year-olds at initial test act as control group to 3-year-olds at testing after 1 year when they are 4; 5-year-olds at initial test act as control group to 4-year-olds at testing after 1 year when they are 5). When comparing these 3-year-olds' reactions after 1 year, they show significantly more correct answers (66%) compared to 4-year-olds before intervention (55% correct, *p* < .044). Similarly, 4-year-olds after 1 year when they turned 5 demonstrate 76% correct answers vs. 64% correct answers in 5-year-olds before intervention start (*p* < .025). These significant differences between the control and intervention groups indicate that the intervention is successful and causes a significant increase in learning.

#### Correct answers and errors – adults

**Figure 6:**
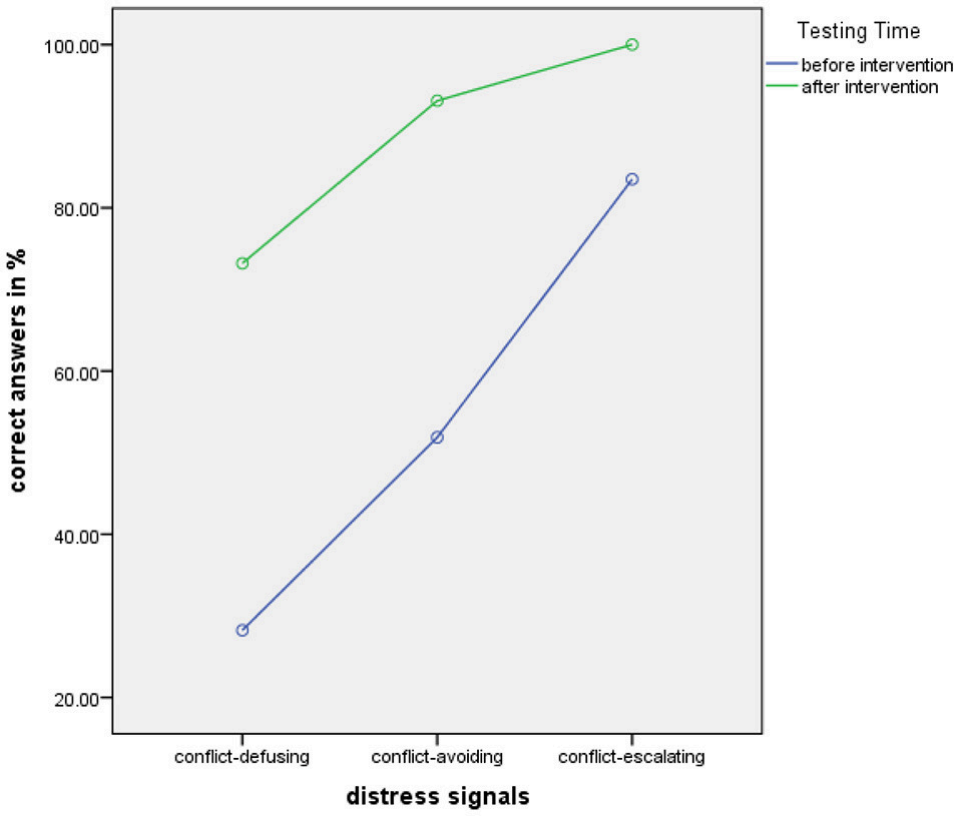
Percent correct scores for adults by distress signal group before and after intervention.

We calculated a repeated measures Anova of Gender (male/female) by Dog Ownership (yes/no) by Distress group (conflict-defusing, conflict-avoiding, conflict-escalating) for Testing Times before and after intervention on percentage of correct answers. Gender and Dog Ownership yielded no significant results, therefore the analysis was calculated with Distress Signal group (conflict-defusing, conflict-avoiding, conflict-escalating) and Testing Times (before and after intervention) on percent correct responses. We found a highly significant main effect for Testing Times [*F*_(1, 39)_ = 311.49, *p* = .0001, partial η^2^ = .89] with better results overall after intervention and a highly significant main effect for Distress Signal Group showing differences between distress signal groups are perceived [*F*_(2, 78)_ = 173.73, *p* = .0001, partial η^2^ = .82]. A highly significant interaction between Testing Time and Distress Signal also emerged [*F*_(2, 78)_ = 26.01, *p* = .0001, partial η^2^ = .40] demonstrating higher rates of correct answers with higher distress as well as rates of correct answers rising from conflict-defusing via avoiding to escalating and all scores being higher after intervention. Results in overview in Figure 6 below.

Interestingly, if we calculate results on a stricter criterium, i.e., only count as correct for conflict-escalating those answers that said “very unhappy/very angry,” all main effects and interactions stay intact, however, performance of adults drops in the conflict-avoiding category to 40% - and in children to 35, 51-, and 60% respectively for 3-, 4-, and 5-year-olds after intervention.

### Additional observations–children's initial perceptions

In addition to the quantitative data described above, we also would like to provide some additional observations. While we were working with the children, they often commented on the videos. The quotes below give an impression of children's thinking and reflect the most frequent comments, see Table 3 below.

**Table 3:** Dog signaling behaviors and children's perceptions and interpretations.

**Dog signaling**	**Child's perception and interpretation**
Dog growling/snarling	“Is really happy and makes a funny noise!” “I could go and cuddle and kiss it - it is so happy!”
Dog staring/stiffening up	“It's looking at me – it likes me!”
Dog lying down, legs up	“It wants me to tickle its belly!”
Dog crouching, tail tucked	“It's sad – let me go and cuddle it to cheer it up!”
Dog hiding under couch	“Surely, dog wants to play hide and seek!”
Dog is yawning	“Must be tired!”
Dog shows nose/lip-licking	“Something tasty on its nose”

These comments were frequently made and show that children often anthropomorphise dogs and try to find an explanation that would be appropriate to explain human behavior, but unfortunately does not fit the dog's signaling intentions.

We would furthermore like to report, so far also only anecdotal comments of parents stating that they frequently provoked distress-signaling behaviors, for example, like lip/nose-licking in their dog, as the family found it funny. However, having learned about dogs' distress signaling in the intervention, the adults were upset that they and their children might have caused their dog distress and commented that they will change their (and their children's) behavior, thus contributing to a safer home environment for all and to dogs' welfare. Further research will need to be carried out to investigate this systematically.

## Discussion

Results show that children and adults profit from the intervention and improve their knowledge of dogs' stress signaling significantly. When performing analyses over time we found that, overall, learning effects are still highly significant in children after 6 months and 1 year despite no training taking place in the meantime–thus, the intervention works successfully, even over the duration of 1 year.

A closer look at the error results shows us the areas in which the intervention has worked most successfully, and also the areas in which we need to invest more training with children and parents alike. We have very good success teaching all age groups of children, even young children of 3 years, and parents the meaning of conflict-escalating distress signals. They learn to understand, recognize and correctly interpret the signals and the learning success is still evident after 1 year. This is an important success as dogs showing their teeth or snarling or biting, pose a significant risk to children if these approach the dogs displaying such signaling. We have good to moderate success in training especially older (5-year-old) children and parents on conflict-avoiding distress signals. However, the data also show that all participants, including adults, find the more subtle signals of dogs' distress hardest to judge. Here, after intervention, only adults show excellent improvements. More research is needed to analyse these signals and how they are perceived in detailed examinations of this in future studies.

One could also question whether children's increase in knowledge is due to general learning and increase in maturity–however, the results of the 4- and 5-year-olds clearly contradict this as children who have taken part in the interventions (3-and 4-year-olds tested after 1 year when they turned 4 and 5 respectively) show significantly better results than the 4-and 5-year-olds at the start of the study (before intervention). Thus, our intervention has clearly improved their knowledge over time compared to the control group. To investigate the role of the intervention in light of children's learning and general maturity over time, it could be useful to devise larger studies with independent control groups, hence requiring significantly larger funding sources.

Overall, it becomes evident from this data that it is possible to educate adults and children to understand dogs' distress signaling. Adults profited from the intervention throughout all distress categories and show clear and significant learning effects. Thus, it is advisable to teach dog signaling to parents, dog owners, dog trainers, veterinary students and the wider public. The short intervention is easy to use and leads to significant improvements in knowledge, recognition and interpretation straight away and with enduring effect.

It has also become clear which areas need further attention and research–while our intervention works very well with adults and also with older children, it has to be adapted to improve especially the younger children's understanding, especially of the more subtle distress signals in dogs. Further research will need to explore how children process the signals and how to teach these signals best.

Our background measures of dog ownership, SES/parental education showed that there were no effects of any of these factors–in other words, neither children's nor parents' performance was better if, for example, they owned a dog, had a higher SES/education. Instead, performance was independent of these factors.

There was also no difference between children seeing novel stimuli in all test phases or the same stimuli again. This is useful to know for the future creation of interventions as we can now be confident that we do not need to increase the amount of novel stimuli to be shown in order to train and assess children on dog body signaling.

Finally, children's utterances illustrated how they perceived—and misinterpreted—dogs' body language. Further quantitative as well as qualitative research in this area is warranted and could help develop additional dog bite prevention tools.

By assessing if our intervention works, we have undertaken the first step toward preventing misunderstandings and risk escalation due to addressing the current lack of knowledge and replacing it with knowledge that is stable over time. In the case of conflict-escalating signals, all participants showed significant improvements in knowledge over time.

Further steps next to teaching children and parents *Knowledge* of stress signaling (step 1) and *Recognition and correct interpretation* of stress signaling in context (step 2) are to *Adapt the action. H*aving created awareness of the situation, insight to act accordingly should follow (step 3). Finally, *Repeat recognition* of future triggers and contexts and avoidance of risk (step 4) need to follow to effectively implement the taught knowledge. Further research will have to assess how to achieve these aims best.

In particular, future studies should address how best to implement the above so that beyond recognizing and understanding the signals, specific human actions and contexts wherein the dog presents these signs are recognized. It will also be useful to investigate if parents—or other educators—can guide and educate children to be aware of specific risk contexts. Concerning parental supervision, it would be interesting to find out to what extent they supervise child and dog and stop children from engaging in risky contexts with their dog in the first place. Furthermore, it would be beneficial to follow up in how far the welfare of family and dog are compromised after escalations have happened as well as investigate the role of professional help from a veterinary behavioral specialist.

Finally, and importantly, we assume that dogs will benefit from children and adults having been taught how to read their distress signals. This increased understanding will mean that dogs are better understood, and if humans apply their knowledge appropriately this will lead to greater wellbeing of the dog living within a family household.

## Conclusion

This project is the first to offer an intervention to enhance children's and adults' abilities to interpret dog signaling correctly.

We investigated how children perceive and categorize dogs' body language and interpret their signals and we then trained them and were able to improve their knowledge, recognition and interpretation skills.

We showed very good results in improving the potentially very dangerous misunderstandings of dogs' conflict-escalating distress and threat signals. For example, a snarling dog showing teeth which children often misinterpreted as a happy dog, can now be corrected–children showed significant improvements that were stable over time. We have shown successfully that we can significantly improve all participants' abilities to recognize and understand these signals and enable all participant groups to avoid escalating risk situations–our intervention works especially well for these high risk situations. This is especially useful as—if such escalation occurs—it should be stopped to avoid risk of dog bite incidents and continued stress to the dog. Crucially, as our intervention furthers understanding of conflict-defusing and conflict-avoiding signals, hopefully, this may help to avoid risk escalation.

We have revealed the extent of children's and adults misinterpretation errors for the first time, and we have shown areas in which children and adults make most errors. We have also shown that we can teach adults and children successfully to learn, recognize and interpret the signals correctly.

With this new knowledge we enhance the currently scarce scientific database on children's and adults' interpretation abilities of dog signaling. We can now also address not only the most dangerous misinterpretations, but also commit ourselves to creating awareness of the less well understood and most frequently misunderstood signaling behaviors of dogs in order to avoid escalation of risk. The materials used can be further developed into an awareness raising intervention that is more widely usable for children and adults. For future effective prevention the above mentioned steps of implementation need to follow and, in turn, also be assessed as to their effectiveness.

In sum, we have now got a solid knowledge base about how children and adults look at and perceive dogs and (mis)interpret their behavior.

Our study was able to close these particular knowledge gaps, establish the necessary knowledge for the first time and therefore significantly advance the scientific knowledge in this area. Our study was also able to show that we can teach dog signaling successfully, and it outlines the current limitations.

Veterinarians will profit from these results insofar as they can help to raise awareness of the existing knowledge gaps in both adults and children.

Our study can also serve as an example of good practice in that we have evaluated the learning effects of the intervention cross-sectionally and longitudinally, as well as using additional measures.

In the future, integrated research projects including child psychology, veterinary, medical, educational and other social sciences can be developed as a result of these efforts and produce research with impact on One Health-related injury prevention challenges.

## Author contributions

KM conceived and designed the research project. TD fed back on the project proposals, contributed to their improvement and contributed the majority of dog videos. As behavior specialist, she also carefully assessed and commented on the video pool and helped select appropriate videos. KM and VB contributed to all aspects of the research itself from planning to testing to data analysis and writing up results. TD also contributed to initial design and planning and to writing the manuscript.

### Conflict of interest statement

The authors declare that the research was conducted in the absence of any commercial or financial relationships that could be construed as a potential conflict of interest.

## Footnotes

^1^Incidentally, our behavior experts agreed least on “happy” dogs. In order to teach about relaxed dog behavior, we would need to set up a separate study investigating this. For the current research, we analyzed the behaviors that were trained in the intervention to see if we can educate participants on recognizing distress behaviors in dogs.

^2^See Norman (105) and Carifio and Perla (106) for the appropriateness of using Likert-scale data with ANOVAs.

^3^For the purpose of scoring % correct, we have scored “unhappy/angry”(4) as correct for conflict-defusing distress, and have accepted both, “unhappy/angry” (4) and “very unhappy/very angry”(5), as correct for conflict-avoiding distress and conflict-escalating distress. In a stricter analysis below, we have only accepted “very unhappy/very angry” (5) as correct for highly distressed dogs. Here, we have accepted both 4 and 5 for highly distressed dogs (instead of just accepting 5s) due to adults known reluctance to give extreme measures for emotional stimuli [e.g., (107)].
